# Iron, coronary artery calcification, and mortality in patients undergoing hemodialysis

**DOI:** 10.1080/0886022X.2021.1880937

**Published:** 2021-02-18

**Authors:** Sonoo Mizuiri, Yoshiko Nishizawa, Toshiki Doi, Kazuomi Yamashita, Kenichiro Shigemoto, Koji Usui, Michiko Arita, Takayuki Naito, Shigehiro Doi, Takao Masaki

**Affiliations:** a Division of Nephrology, Ichiyokai Harada Hospital, Hiroshima, Japan; b Department of Nephrology, Hiroshima University Hospital, Hiroshima, Japan; c Ichiyokai Ichiyokai Clinic, Hiroshima, Japan; d Iciyokai East Clinic, Hiroshima, Japan; e Ichiyokai Yokogawa Clinic, Hiroshima, Japan

**Keywords:** Coronary artery calcification, hemodialysis, iron, mortality

## Abstract

**Objective:**

A high coronary artery calcification score (CACS) may be associated with high mortality in patients undergoing hemodialysis (HD). Recently, effects of iron on vascular smooth muscle cell calcification have been described. We aimed to investigate the relationships between iron, CACS, and mortality in HD patients.

**Methods:**

We studied 173 consecutive patients who were undergoing maintenance HD. Laboratory data and Agatston’s CACS were obtained at baseline for two groups of patients: those with CACS ≥400 (*n* = 109) and those with CACS <400 (*n* = 64). Logistic regression analyses for CACS ≥400 and Cox proportional hazard analyses for mortality were conducted.

**Results:**

The median (interquartile range) age and duration of dialysis of the participants were 67 (60–75) years and 73 (37–138) months, respectively. Serum iron (Fe) and transferrin saturation (TSAT) levels were significantly lower in participants with CACS ≥400 than in those with CACS <400, although the serum ferritin concentration did not differ between the groups. TSAT ≥21% was significantly associated with CACS ≥400 (odds ratio 0.46, *p<*0.05). TSAT ≥17%, Fe ≥63 µg/dL, and ferritin ≥200 ng/mL appear to protect against 5-year all-cause mortality in HD patients, independent of conventional risk factors of all-cause mortality (*p* < 0.05).

**Conclusion:**

We have identified associations between iron, CACS, and mortality in HD patients. Lower TSAT was found to be an independent predictor of CACS ≥400, and iron deficiency (low TSAT, iron, or ferritin) was a significant predictor of 5-year all-cause mortality in HD patients.

## Introduction

Vascular calcification (VC) is recognized to be one of the main causes of cardiovascular disease in patients with end-stage renal disease [1]. Iron deficiency is common in patients with advanced kidney disease, and particularly in those requiring hemodialysis (HD) [2]. Whereas the effect of excess iron to promote VC via the generation of reactive oxygen species is well recognized [3], the optimal dose of iron to be administered in HD patients has been a major point of debate among nephrologists.

Recently, data regarding the effect of iron on vascular smooth muscle cell (VSMC) calcification have been published [4–8]. Iron treatment reduces aortic medial calcification in uremic rats [4] and iron citrate reduces high phosphate-induced VC by inhibiting apoptosis [5] and osteo-chondrogenic shift in VSMCs [6]. Ferritin has been reported to prevent calcification and the osteoblastic transformation of smooth muscle cells [7,8]. Balla et al. [9] proposed that the mechanism of the inhibition of phosphate-induced VSMC calcification involves 3H-1,2-dithiole-3-thione and iron-induced upregulation of the ferritin heavy chain. A high coronary artery calcification score (CACS ≥400) may be associated with higher mortality in HD patients [10,11]. In the general population, the CACS indicates the degrees of atherosclerosis and intimal calcification, but in HD patients it principally indicates the degree of medial wall calcification [12]. Studies of the effect of iron on VSMC transformation [6–9] suggest that it is associated with medial wall calcification, and therefore the circulating iron concentration may be associated with the severity of VC and the mortality of patients undergoing HD. We aimed to investigate the relationships between iron, CACS, and mortality in HD patients.

## Materials and methods

### Patients

We conducted a retrospective observational study of 173 consecutive patients who were undergoing maintenance HD (MHD) at our hospital. The study began in September 2012, and the observation period started at study enrollment and ended at the first one of the following: death, transfer to another facility, or the end of study (September 2017; 5 years after study enrollment began). The exclusion criteria were as follows: <20 years of age, duration of dialysis <3 months, history of advanced cancer, infection in the month prior to potential enrollment, and organ transplantation.

### Procedures

All the participants had vascular access that provided a blood flow rate of ≥200 mL/min and underwent 4 h of HD or 4 h of predilution on-line hemodiafiltration (HDF) with a total convective volume of 40 L per session, three times per week. A standard bicarbonate dialysis fluid, containing 140 mEq/L sodium, 2.0 mEq/L potassium, 3.0 mEq/L calcium, 1.0 mEq/L magnesium, and 100 mg/dL glucose was delivered using a central dialysis fluid delivery system during HD and on-line HDF.

The baseline data for the age, sex, type of primary renal disease, presence of diabetes mellitus, and duration of dialysis, the survival time, and the cause of death for each participant were obtained from the institutional database. The blood pressure; hemoglobin (Hb); transferrin saturation (TSAT); and the serum iron (Fe), ferritin, albumin, C-reactive protein (CRP), phosphate, intact parathyroid hormone, intact fibroblast growth factor 23 (FGF23), and albumin-adjusted serum calcium (Ca) concentrations of each participant were measured just before their first dialysis session of the week. Furthermore, their serum urea concentration was measured just before and after the first dialysis session of the week and just before the next dialysis session. Serum intact FGF23 concentrations were determined by SRL (Tokyo, Japan) using sandwich ELISA kits (Kainos, Tokyo, Japan). Other biochemical analyses were performed in the hospital laboratory and the normal ranges for the general population of TSAT, Fe, and ferritin were 20–40%, 80–200 µg/dL, and 21–282 ng/mL, respectively.

Three types of erythropoiesis-stimulating agents (ESA): epoetin beta, darbepoetin alfa, and epoetin beta pegol were used in the study, and the dose of ESA was converted to that of epoetin using the dose conversion ratio epoetin beta: darbepoetin alfa: epoetin beta pegol of 200:1:0.93 [13]. Erythropoietin responsiveness index (ERI) was defined as the average weekly ESA dose divided by dry weight and average blood hemoglobin (weekly ESA dose (units)/dry weight (kg)/hemoglobin (g/dL) as described previously [14]. The prescribed doses of active vitamin D and phosphate-binding agents were converted to daily defined doses (DDDs), using the conversion factors provided by the World Health Organization Drug Classification (http://www.whocc.no/atcddd/), and they are expressed as prescribed weekly dose/7 DDD value. The phosphate binders used were calcium carbonate (DDD: 3 g), sevelamer (DDD: 6.4 g), and lanthanum carbonate (DDD: 2.25 g). Active vitamin D was administered in the form of calcitriol (DDD: 1 µg) or alfacalcidol (DDD: 1 µg). Other phosphate binders and active vitamin D agents were not prescribed at our institution in 2012. The baseline doses of cinacalcet, oral iron [sodium ferrous citrate (Fe^2+^) (Ferromia^Ⓡ^; Eisai Co. Ltd. Tokyo, Japan)] [15], or intravenous iron [saccharated ferric oxide (Fesin^Ⓡ^, Nichi-Iko Pharmaceutical, Toyama, Japan)] were also recorded. All-cause mortality during the follow-up period was determined using the hospital records. The CACSs were determined at baseline using the Agatston’s CACS [16], based on the results of thoracoabdominal multidetector computed tomography, performed using an Aquilion 64 TSX-101A (Toshiba Medical Systems, Tokyo, Japan) at baseline.

### Endpoints

The primary endpoint of the study was a CACS ≥400 and the secondary endpoint was 5-year all-cause mortality.

### Statistical analyses

Statistical analyses were performed using JMP13 (SAS Institute Japan, Tokyo, Japan). The Kolmogorov-Smirnov test was used to determine whether datasets were normally distributed. Categorical data are reported as numbers of participants (percentages) and continuous data are reported as means ± standard deviations (SDs) or medians [interquartile ranges (IQRs)], as appropriate. MHD patients were placed into two groups according to their baseline CACS: ≥400 or <400. Datasets were compared between the two groups using the Wilcoxon signed-rank test if continuous and Fisher’s exact test if categorical. Logistic regression analysis was used to identify factors that were associated with CACS ≥400 in the full sample. Cox proportional hazard analyses were performed to identify factors that were associated with 5-year all-cause mortality. The distribution of intact FGF23 was markedly skewed; therefore, prior to the analysis, intact FGF23 was transformed to log intact FGF23. The Kaplan-Meier 5-year cumulative survival curves of the two groups were compared. Cutoff values for TSAT, Fe, and ferritin for CACS ≥400 and 5-year all-cause mortality were determined using receiver operating characteristic (ROC) analysis and the area under the ROC curve (AUC). Additionally, Spearman’s rank correlations between CRP and markers of iron status were calculated.

### Ethics statement

The Committee on Human Research at Ichiyokai Hospital approved the study protocol (authorization number 202005), which conformed to the provisions of the Declaration of Helsinki, as revised in Brazil in 2013. The requirement for informed consent was waived by the ethics committee because we used de-identified retrospective data that had been collected during routine patient management.

## Results

In the 173 HD patients, the primary renal diseases were chronic glomerulonephritis (*n* = 82, 47.4%), diabetic nephropathy (*n* = 63, 36.4%), nephrosclerosis (*n* = 16, 9.3%), polycystic kidney disease (*n* = 4, 2.3%), other diseases (*n* = 7, 4.0%), and unknown conditions (*n* = 1, 0.6%). The clinical characteristics of all the participants and of the two groups that were defined according to their CACS (≥400, *n* = 109; and <400, *n* = 64) are shown in Table 1. The median (IQR) age and duration of dialysis of the complete sample of patients were 67 (60–75) years and 73 (37–138) months, respectively. The median (IQR) CACS for all the participants was 1,025 (190–2,832). As shown in Table 1, participants with CACS ≥400 were significantly older (*p<*0.01), had been undergoing dialysis for longer (*p<*0.01), had higher prevalence of previous and current cardiovascular disease (*p<*0.01), and had lower TSAT (*p<*0.05), lower Fe (*p<*0.05), higher serum CRP (*p<*0.001), and higher serum Ca (*p<*0.05) concentrations than those with CACS <400. The sex, prevalence of diabetes, prevalence of smoking, blood pressure, Kt/Vurea, hemoglobin, and serum concentrations of ferritin, albumin, phosphate, intact parathyroid hormone, and intact FGF23 did not differ significantly between the two groups. The ERI, dose of ESA, frequency of oral or intravenous iron use, dose of oral iron, dose of all active vitamin D3, dose of calcium carbonate, dose of all phosphate binders, dose of cinacalcet, frequency of statin use, and frequency of renin-angiotensin system (RAS) inhibitor use also did not significantly differ between the groups, and intravenous iron was administered to only one patient with CACS ≥400, at 40 mg/week.

**Table 1.** Baseline characteristics of hemodialysis patients, grouped according to their CACS.

Characteristics	All	CACS ≥400	CACS <400	*p*
	*n* = 173	*n* = 109	*n* = 64	
CACS	1,025 (190–2832)	2,456 (1208–3869)	131 (10–309)	<0.0001
Age (years)	67 (60–75)	70 (6–77)	62 (52–71)	<0.01
Male sex *n* (%)	121/173 (69.9)	77/109 (70.6)	44/64 (68.8)	0.86
Duration of dialysis (months)	73 (37–138)	90 (50–149)	55 (31–100)	<0.01
Presence of diabetes mellitus n (%)	73,173 (36.4)	43/109 (39.5)	20/64 (31.3)	0.33
Presence of cardiovascular disease	134/173 (77.5)	93/109 (85.3)	41/64 (64.1)	<0.01
Smoking n (%)	81/173 (46.8)	49/109 (45.0)	32/64 (50.0)	0.53
Systolic blood pressure (mmHg)	153 ± 24	156 ± 24	148 ± 24	0.08
Diastolic blood pressure (mmHg)	81 ± 14	80 ± 14	83 ± 13	0.25
Kt/Vurea (/dialysis session)	1.40 (1.27–1.50)	1.40 (1.27–1.49)	1.40 (1.27–1.52)	0.68
Hemoglobin (g/dL)	11.4 (10.6–12.3)	11.4 (10.5–12.3)	11.5 (10.8–12.3)	0.27
TSAT (%)	23 (16–31)	21 (14–28)	26 (19–34)	<0.05
Fe (μg/dL)	60 (41–82)	56 (39–77)	66 (50–89)	<0.05
Ferritin (ng/mL)	36.6 (23.0–72.5)	37.8 (24.6–69.2)	33.9 (22.4–27.3)	0.88
Serum albumin (g/dL)	3.4 (3.2–3.7)	3.6 (3.4–3.8)	3.7 (3.4–4.0)	0.22
Serum CRP (mg/dL)	0.13 (0.04–0.43)	0.21 (0.07–0.46)	0.06 (0.03–0.22)	<0.001
Albumin-adjusted serum calcium (mg/dL)	9.4 ± 0.7	9.5 ± 0.7	9.3 ± 0.6	<0.05
Serum phosphate (mg/dL)	5.0 ± 1.3	5.1 ± 1.3	4.9 ± 1.2	0.67
Serum intact parathyroid hormone (pg/mL)	103 (44–204)	99 (32–175)	112 (55–224)	0.12
Serum intact FGF23 (pg/mL)	1,930 (473–5555)	2,020 (473–5730)	1,770 (485–4955)	0.93
Erythropoietin responsiveness index (unit/kg/g/dL)	7.0 (4.6–12.7)	7.6 (4.6–13.3)	6.0 (4.5–11.4)	0.14
Dose of ESA (unit/week)	4,000 (3000–8000)	6,000 (3000–8000)	4,000 (3000–7500)	0.14
Frequency of oral or intravenous iron use n (%)	109/173 (63.0)	66/109 (60.6)	43/64 (67.2)	0.38
Dose of oral iron (mg/day)	21.4 (0–50)	21,4 (0–23.2)	21.4 (0–50)	0.31
Dose of all active vitamin D3 (μg/day)*	0.21 (0–0.50)	0.21 (0–0.50)	0.25 (0–0.71)	0.40
Dose of calcium carbonate (g/day)	1.5 (0.0–3.0)	1.5 (0.0–3.0)	1.5 (0.6–3.0)	0.11
Dose of all phosphate binders (g/day)*	0.67 (0.42–1.33)	0.67 (0.33–1.27)	0.83 (0.50–1.41)	0.26
Dose of cinacalcet (mg/day)	0 (0–0)	0 (0–0)	0 (0–0)	0.18
Statin use *n* (%)	11/173 (6.4)	8/109 (7.3)	3/64 (4.7)	0.48
RAS inhibitors use *n* (%)	104/173 (60.1)	65/109 (59.6)	39/64 (60.9)	1.00

CACS: coronary artery calcification score; TSAT: transferrin saturation; Fe: serum iron; ferritin: serum ferritin; FGF23: fibroblast growth factor 23; ESA: erythropoiesis stimulating agent; Erythropoietin responsiveness index was defined as the average weekly ESA dose divided by dry weight and average blood hemoglobin. RAS: renin-angiotensin system.

*The prescribed doses of active vitamin D and phosphate-binding agents were converted to daily defined doses (DDDs), using the conversion factors provided by the World Health Organization Drug Classification (http://www.whocc.no/atcddd/), and they are expressed as prescribed weekly dose/7 DDD value. The phosphate binders used were calcium carbonate (DDD: 3 g), sevelamer (DDD: 6.4 g), and lanthanum carbonate (DDD: 2.25 g). Active vitamin D was administered in the form of calcitriol (DDD: 1 µg) or alfacalcidol (DDD: 1 µg). Other phosphate binders and active vitamin D agents were not prescribed at our institution in 2012.

ROC curves were generated using the conventional risk factors for CACS (age, sex, smoking, presence of diabetes mellitus, duration of dialysis, serum Ca, phosphate, CRP, and ERI) +TSAT, the conventional risk factors for CACS + Fe, or the conventional risk factors for CACS + ferritin, for the prediction of CACS ≥400 (*n* = 173) (Supplementary Figure 1). For the conventional risk factors for CACS + TSAT, the AUC was 0.766 (0.684–0.832), and the cutoff value of TSAT was 21%, which was associated with a sensitivity of 76% and a specificity of 67%. For the conventional risk factors for CACS + Fe, the AUC was 0.763 (0.680–0.831) and the cutoff value of Fe was 72 µg/dL, which was associated with a sensitivity of 73% and a specificity of 72%. For the conventional risk factors for CACS + ferritin, the AUC was 0.769 (0.685–0.836) and the cutoff value of ferritin was 155 ng/mL, which was associated with a sensitivity of 79% and a specificity of 64%.

The results of logistic regression analysis of all the participants, which aimed to identify factors associated with CACS ≥400, are shown in Table 2. In the univariate logistic regression analysis, CACS ≥400 was significantly associated with age, duration of dialysis, TSAT ≥21%, and Fe ≥72 µg/dL (*p<*0.05), but there were no associations with other variables. In the multivariate analysis that included conventional risk factors (Model 1), age (*p<*0.0001), the presence of diabetes mellitus (*p<*0.05), and the duration of dialysis (*p<*0.05) were significantly associated with CACS ≥400 in HD patients. When TSAT ≥21% was added to Model 1 (Model 2), TSAT ≥21% was significantly associated with CACS ≥400, independent of the conventional risk factors for CACS *(p<*0.05). When Fe ≥72 µg/dL was added to Model 1 (Model 3), Fe ≥72 µg/dL was not found to be a significant predictor of CACS ≥400 in HD patients. When ferritin ≥155 ng/mL was added to Model 1 (Model 4), ferritin ≥155 ng/mL was not found to be a significant predictor of CACS ≥400 in HD patients.

**Table 2.** Factors associated with CACS ≥400 in hemodialysis patients (*n* = 173).

Variables	Univariate analyses			Multivariate analyses											
				Model 1			Model 2			Model 3			Model 4		
	OR	95% CI	*p*	OR	95% CI	*p*	OR	95% CI	*P*	OR	95% CI	*p*	OR	95% CI	*p*
Age (years)	1.06	1.03–1.09	<0.0001	1.07	1.04–1.10	<0.0001	1.07	1.04–1.10	<0.0001	1.07	1.03–1.10	<0.0001	1.07	1.04–1.10	<0.0001
Male sex	1.09	0.56–2.14	0.79	1.24	0.54–2.84	0.61	1.30	0.56–3.04	0.54	1.39	0.59–3.26	0.45	1.18	0.51–2.72	0.70
Smoking	1.22	0.66–2.27	0.52	1.04	0.49–2.21	0.92	1.10	0.51–2.37	0.82	1.15	0.53–2.49	0.73	1.01	0.47–2.17	0.98
Presence of diabetes mellitus	1.43	0.75–2.75	0.28	2.23	1.02–4.86	<0.05	2.35	1.07–5.17	<0.05	2.20	1.01–4.81	<0.05	1.01	1.00–1.01	<0.05
Duration of dialysis (months)	1.01	1.00–1.01	<0.05	1.01	1.00–1.01	<0.01	1.01	1.00–1.01	<0.01	1.01	1.00–1.01	<0.01	1.01	1.00–1.01	<0.01
Adjusted serum calcium (mg/dL)	1.58	0.97–2.56	0.06	1.38	0.80–2.37	0.24	1.42	0.82–2.45	0.21	1.42	0.82–2.46	0.21	1.44	0.83–2.51	0.19
Serum phosphate (mg/dL)	1.10	0.86–1.41	0.45	1.26	0.95–1.69	0.11	1.29	0.96–1.73	0.10	1.32	0.98–1.79	0.06	1.27	0.95–1.71	0.10
Serum CRP (mg/dL)	1.37	0.78–2.37	0.27	1.26	0.69–2.32	0.45	1.15	0.65–2.04	0.62	1.16	0.64–2.08	0.62	1.26	0.66–2.42	0.49
Erythropoietin responsiveness index	1.01	0.97–1.04	0.76	1.00	0.96–1.04	0.97	1.00	0.96–1.04	0.98	1.00	0.96–1.04	0.95	1.00	0.95–1.03	0.72
TSA*T* ≥ 21%	0.46	0.24–0.90	<0.05				0.46	0.22–0.96	<0.05						
F*e* ≥ 72 (µg/dL)	0.47	0.25–0.89	<0.05							0.51	0.24–1.07	0.08			
Ferriti*n* ≥ 155 (ng/mL)	0.63	0.21–1.84	0.39										0.42	0.11–1.61	0.21
Log serum intact FGF23 (pg/mL)	0.95	0.62–1.45	0.81												
Dose of ESA (unit/week)	1.00	0.99–1.00	0.15												
Dose of oral iron (mg/day)	0.99	0.98–1.01	0.42												

CACS: coronary artery calcification score; CRP: C-reactive protein; TSAT: transferrin saturation; Fe: serum iron; ferritin: serum ferritin; FGF23: fibroblast growth factor 23; ESA: erythropoiesis stimulating agent; Erythropoietin responsiveness index (unit/kg/g/dL) was defined as the average weekly ESA dose divided by dry weight and average blood hemoglobin (weekly ESA dose (units)/dry weight (kg)/hemoglobin (g/dL).

OR: odds ratio, CI: confidence interval, For continuous variables, the ORs were computed per unit increase.

The observation period was 5 years for all the participants. During this time period, of the 173 participants, 57 died and 6 were transferred to another facility. The causes of death were infection (20/57; 35.0%), heart failure (12/57; 21.1%), cerebrovascular disease (5/57; 8.8%), myocardial infarction (3/57; 5.3%), malignancy (2/57; 3.5%), others (10/57; 17.5%), and unknown (5/57; 8.8%). ROC curves were generated using the conventional risk factors for all-cause mortality (age, sex, smoking, presence of diabetes mellitus, duration of dialysis, CACS, serum albumin, CRP, log intact FGF23, ERI, and the dose of calcium carbonate) + TSAT, the conventional risk factors for all-cause mortality + Fe, or the conventional risk factors for all-cause mortality + ferritin, for the prediction of 5-year all-cause mortality (*n* = 173) (Supplementary Figure 2). For the conventional risk factors for all-cause mortality + TSAT, the AUC was 0.885 (0.777–0.945) and the cutoff value of TSAT was 17%, which was associated with a sensitivity of 77% and a specificity of 85%. For the conventional risk factors for all-cause mortality + Fe, the AUC was 0.883 (0.777–0.943) and the cutoff value of Fe was 63 µg/dL, which was associated with a sensitivity of 77% and a specificity 84%. For the conventional risk factors for all-cause mortality + ferritin, the AUC was 0.853 (0.730–0.926) and the cutoff value of ferritin was 200 ng/mL, which was associated with a sensitivity of 77% and a specificity of 86%.

The predictors of 5-year all-cause mortality for all the participants, according to Cox proportional hazard analyses, are shown in Table 3. In the univariate analyses, age, duration of dialysis, CACS, serum albumin, CRP, log intact FGF23, ERI, dosage of calcium carbonate, and Fe ≥63 µg/dL were significant predictors of 5-year all-cause mortality. In the multivariate analysis that included the conventional risk factors for all-cause mortality (age, sex, smoking, presence of diabetes mellitus, duration of dialysis, CACS, serum albumin, CRP, log intact FGF23, ERI, and dose of calcium carbonate) (Model 1), the age, CACS, serum CRP, ERI, and dose of calcium carbonate were significant predictors of 5-year all-cause mortality. When TSAT ≥17% was added to Model 1 (Model 2), TSAT ≥17% was a significant predictor of 5-year all-cause mortality, independent of the conventional risk factors. When Fe ≥63 µg/dL was added to Model 1 (Model 3), Fe ≥63 µg/dL was a significant predictor of 5-year all-cause mortality, independent of the conventional risk factors. When ferritin ≥200 ng/mL was added to Model 1 (Model 4), ferritin ≥200 ng/mL was a significant predictor of 5-year all-cause mortality, independent of the conventional risk factors. Furthermore, ERI was a significant predictor of 5-year all-cause mortality in all the models (Model 1–4). When the dose of ESA was switched for ERI in Model 1 (Model 5, data not shown), the age, duration of dialysis, CACS, serum CRP, dose of ESA, and dose of calcium carbonate were significant predictors of 5-year all-cause mortality (*p<*0.05). The predictors of 5-year all-cause mortality in HD patients, according to Cox proportional hazard analyses using the dose of all phosphate binders as a variable, are provided in the Supplementary Table. The dose of all phosphate binders (calcium carbonate + sevelamer + lanthanum carbonate) was a significant predictor of 5-year all-cause mortality in all the models (Supplementary Table, Models 1–3).

**Table 3.** Predictors of 5-year all-cause mortality in hemodialysis patients, according to Cox proportional hazard analysis (*n* = 173).

Variables	Univariate analyses			Multivariate analyses											
				Model 1			Model 2			Model 3			Model 4		
	HR	95% CI	*p*	HR	95% CI	*p*	HR	95% CI	*p*	HR	95% CI	*p*	HR	95% CI	*p*
Age (years)	1.10	1.07–1.13	<0.0001	1.07	1.03–1.11	<0.001	1.07	1.03–1.10	<0.001	1.06	1.03–1.10	<0.001	1.06	1.03–1.10	<0.001
Male sex	1.11	0.62–1.90	0.73	1.13	0.61–2.17	0.70	1.06	0.56–2.03	0.87	1.11	0.59–2.13	0.75	1.20	0.64–2.32	0.57
Smoking	1.69	1.00–2.92	0.05	1.23	0.65–2.36	0.53	1.24	0.64–2.42	0.52	1.35	0.71–2.72	0.34	1.39	0.72–2.74	0.33
Presence of diabetes mellitus	1.58	0.93–2.65	0.09	1.32	0.69–2.53	0.40	1.38	0.71–2.64	0.34	1.35	0.70–2.57	0.35	1.15	0.59–2.20	0.68
Duration of dialysis (months)	1.00	0.99–1.00	<0.05	1.00	0.99–1.00	0.32	1.00	0.99–1.00	0.26	1.00	0.99–1.00	0.19	1.00	0.99–1.00	0.14
CACS	1.01	1.00–1.01	<0.0001	1.01	1.00–1.01	<0.0001	1.01	1.00–1.01	<0.001	1.01	1.00–1.01	<0.0001	1.01	1.00–1.01	<0.0001
Serum albumin (g/dL)	0.18	0.10–0.35	<0.0001	0.59	0.28–1.25	0.17	0.58	0.27–1.28	0.17	0.73	0.33–1.62	0.43	0.54	0.24–1.19	0.13
Serum CRP (g/dL)	1.76	1.33–2.19	<0.0001	1.65	1.23–2.23	<0.001	1.70	1.21–2.28	<0.01	1.64	1.15–2.21	<0.01	1.72	1.22–2.32	<0.01
Log serum intact FGF23 (pg/mL)	0.56	0.39–0.79	<0.001	0.89	0.60–1.33	0.57	0.90	0.60–1.35	0.62	0.85	0.56–1.27	0.42	0.85	0.57–1.25	0.39
Erythropoietin Responsiveness Index	1.02	1.00–1.04	<0.01	1.03	1.01–1.05	<0.01	1.04	1.01–1.06	<0.01	1.04	1.01–1.06	<0.01	1.05	1.02–1.08	<0.001
Dose of calcium carbonate (g/day)	0.74	0.61–0.88	<0.01	0.82	0.66–1.00	<0.05	0.80	0.64–0.98	<0.05	0.81	0.65–0.99	<0.05	0.81	0.65–0.98	<0.05
TSA*T* ≥ 17%	0.61	0.36–1.05	0.07				0.54	0.31–0.95	<0.05						
F*e* ≥ 63 (µg/dL)	0.57	0.33–0.96	<0.05							0.53	0.29–0.94	<0.05			
Ferriti*n* ≥ 200 (ng/mL)	0.84	0.35–2.79	0.75										0.24	0.05–0.80	<0.05
Oral or intravenous iron use	0.70	0.42–1.20	0.19												
Dose of oral iron (mg/week)	0.99	0.98–1.01	0.30												
RAS inhibitors use	0.72	0.43–1.22	0.21												
Statin use	1.76	0.61–4.01	0.26												
Dose of ESA (unit/week)	1.01	1.00–1.01	<0.01												

CACS: coronary artery calcification score; CRP: C-reactive protein; FGF23: fibroblast growth factor 23; ESA: erythropoiesis stimulating agent; Erythropoietin responsiveness index (unit/kg/g/dL) was defined as the average weekly ESA dose divided by dry weight and average blood hemoglobin (weekly ESA dose (units)/dry weight (kg)/hemoglobin (g/dL). TSAT: transferrin saturation; Fe: serum iron; Ferritin: serum ferritin; RAS: renin-angiotensin system; HR: hazard ratio; CI: confidence interval. For continuous variables, the HRs were computed per unit increase.

**Figure 1.**
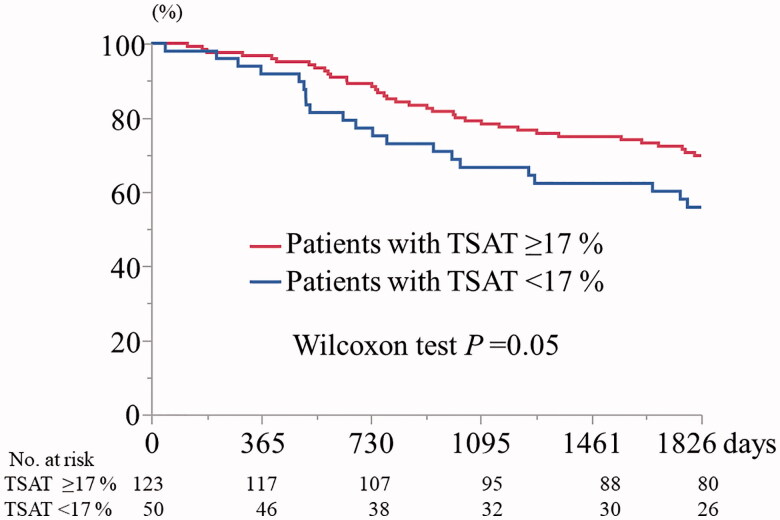
Kaplan-Meier 5-year cumulative survival curves for participants with transferrin saturation ≥17% and <17% (*n* = 173). TSAT: transferrin saturation.

**Figure 2.**
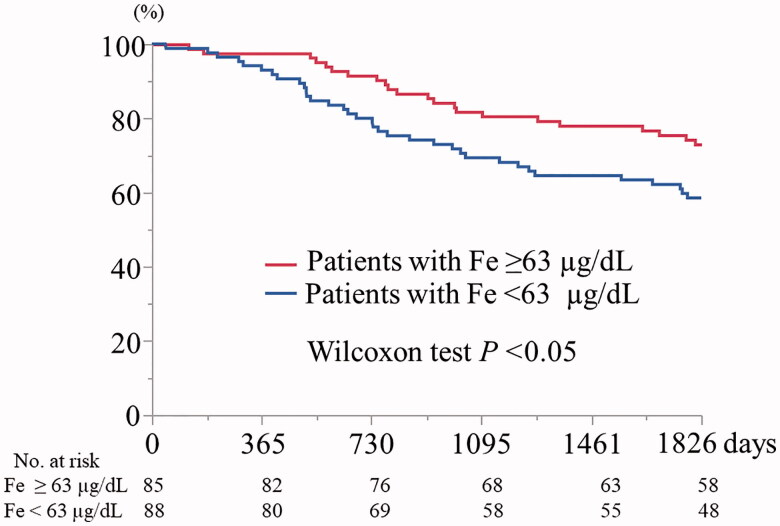
Kaplan–Meier 5-year cumulative survival curves for participants with serum iron ≥63 and <63 µg/dL (*n* = 173). Fe: serum iron.

As shown in Figure 1, the Kaplan-Meier 5-year cumulative survival rates were 70.0% and 55.9% in participants with a TSAT ≥17% and a TSAT <17%, respectively (Wilcoxon test, *p=*0.05). As shown in Figure 2, the Kaplan-Meier 5-year cumulative survival rate for participants with Fe 63 ≥µg/dL was significantly higher than that for participants with Fe <63 µg/dL (73.1% vs. 58.6%, Wilcoxon test, *p<*0.05). Finally, the Kaplan-Meier 5-year cumulative survival rates for participants with ferritin ≥200 ng/mL and with ferritin <200 ng/mL did not significantly differ (*p=*0.67) (data not shown).

A scatterplot matrix of serum CRP, TSAT, Fe, and ferritin for all the participants (*n* = 173) is shown as Supplementary Figure 3. The Spearman’s correlation coefficients for serum CRP and TSAT, serum CRP and Fe, and serum CRP and ferritin were *ρ=*−0.15 (*p=*0.05), *ρ=*−0.27 (*p<*0.001), and *ρ=*0.24 (*p<*0.01), respectively.

## Discussion

In the present study, we found that patients with CACS ≥400 had lower Fe and TSAT levels than patients with CACS <400 and TSAT ≥21% was an independent and significant predictor of CACS <400 in HD patients. TSAT ≥17%, Fe ≥63 µg/dL, and ferritin ≥200 ng/mL were significant predictors of 5-year all-cause mortality in HD patients. We conducted a retrospective observational study, but this represents the first report regarding the relationships between iron, CACS, and mortality in HD patients. Kalantar-Zadeh et al. [17] previously reported that Fe concentrations between 60 and 120 μg/mL and TSAT levels between 30 and 50% are associated with the lowest risks of all-cause and cardiovascular mortality in HD patients. Furthermore, Pollak et al. [18] reported that HD patients lived the longest with values of TSAT of >25% and ferritin of >600 µg/L. Therefore, although it had been reported that Fe, TSAT, and ferritin levels are associated with mortality in HD patients [17,18], the relationship between iron and coronary artery calcification has not been previously evaluated. 

The most common cause of ESA resistance in HD patients is iron deficiency [19] and one previous study showed that the progression of coronary artery calcification is associated with anemia [20]. Consistent with the results of previous studies [21,22], we have shown that ESA hyporesponsiveness is associated with a higher risk of all-cause mortality in HD patients. Taken together, these findings suggest that iron deficiency, iron dose, ESA hyporesponsiveness, coronary artery calcification, and mortality are related in HD patients.

Previous studies have revealed that the hyperphosphatemia caused by chronic kidney disease (CKD) is responsible for the transformation of VSMCs to chondrocytes or osteoblast-like cells and medial calcification [23]. In addition to the possibility that iron increases oxidative stress, which accelerates VC, it is thought that iron might reduce the intracellular phosphate concentration because of its ability to bind phosphate [7,24]. Ferritin preserves iron excess, but counteracts the toxicity of iron, and protects against oxidative stress although some studies have suggested that ferritin can amplify oxidative phenomena [3,25].

Total iron binding capacity (TIBC) is a negative acute-phase reactant and reduction in TIBC induced by inflammation leads to higher TSAT levels independent of iron status, and serum levels of ferritin were positively correlated with CRP, a measure of inflammation in CKD [26]. Inflammation contributes to VC by various mechanisms; for instance, by reducing the circulating fetuin-A concentration [27]. Furthermore, inflammation is associated with a high hepcidin concentration, which increases the ferritin concentration, thereby reducing the free serum iron concentration and TSAT [28]. Patsalas et al. reported that the serum concentration of the inflammatory marker CRP is high in HD patients who have a high CACS [29]. In the present study, we have shown weak correlations between serum CRP and Fe, and serum CRP and ferritin, and no significant correlation was found between serum CRP and TSAT. The associations between serum iron indices and CACS may, at least in part, reflect the impact of inflammation, although serum CRP was not a significant determinant of CACS ≥ 400 in the present study.

Large differences in the management of iron status during the therapy of anemia in HD patients are observed between Western countries and Japan. According to the Japanese Society of Dialysis Therapy (JSDT) 2015 guidelines, oral or intravenous iron therapy is recommended for anemic HD patients on ESA who have TSAT <20% and ferritin <100 ng/mL, although ferritin concentrations of ≥300 ng/mL are not recommended [30]. However, the optimal levels of iron markers for the avoidance of coronary artery calcification are unknown.

The comparative safety of intravenous and oral iron supplementation in CKD patients remains a subject of debate [31]. However, a stronger association was identified between acute reactions and the risk of infectious disease in patients being treated with intravenous iron than that in those being treated with oral iron [30]. Furthermore, it has been reported that intravenous iron administration increases the circulating FGF23 concentration, whereas oral iron reduces FGF23 in these patients [15,32]. Therefore, oral iron is used in preference in our hospital and only one patient had received intravenous iron supplementation at baseline in the present study.

The results of studies of the relationship between FGF23 and the severity of coronary artery calcification have been conflicting [33,34], and in the present study, no significant difference in the intact FGF23 concentration was observed between HD patients with CACS ≥400 and those with < CACS 400. Furthermore, intact FGF23 did not have a significant impact on 5-year all-cause mortality in the present study, in contrast to well-known previous report [35]. However, Olauson et al. [36] reported no association between high serum FGF23 and mortality in HD patients, which is consistent with our findings. The discrepancies between the results of these studies might be at least partially explained by differences in sex, previous cardiovascular diseases, residual renal function [36], and racial differences [35].

The administration of phosphate binders, particularly iron-based phosphate binders [37,38], active vitamin D, and cinacalcet [39] may inhibit the progression of vascular calcification; however, their use remains controversial. Interestingly, iron supplementation when calcification is already present completely prevents further high phosphate induced calcium deposition [5], and TSAT ≥21% was found to be a significant protective predictor of CACS ≥400 in this study. There were no significant differences in the doses of active vitamin D3, iron, calcium carbonate, or phosphate binders (calcium carbonate + sevelamer + lanthanum carbonate) being administered to patients with CACS ≥400 or <400 in the present study. However, the doses of calcium carbonate and phosphate binders in general were both significant predictors of 5-year all-cause mortality in this study. Thus, drugs with phosphate-binding ability appear to reduce mortality in HD patients, whether they are or are not calcium-containing.

The present study had several limitations. First, it was a retrospective, cross-sectional study of a small sample of Japanese people. Second, CACS, Fe, TSAT, ferritin, and FGF23 levels can change over time, and therefore no causal relationships can be inferred from the data. Third, there may be differences in the long-term survival rates that are associated with the use of HD and HDF. However, it was not possible to make a comparison between these two groups in the present study, because it commenced in September 2012, when only 2/173 patients were undergoing HDF. Fourth, all the data were collected at the beginning of the week for all the participants in the study because this is obligatory for all the dialysis units in Japan, to facilitate the annual statistical survey of the JSDT. In contrast, blood is typically collected in the middle of the week in Europe and the United States, and therefore differences in the rate of change of body weight may have influenced the results. Furthermore, the data used to calculate the cutoff values for the markers of iron status may have been affected by differences on the day on which blood sampling occurred.

In conclusion, we have presented evidence for associations between iron, CACS, and mortality in HD patients. Low TSAT was found to be a significant independent predictor of CACS ≥400 and iron deficiency (low TSAT, iron, or ferritin levels) was a significant predictor of 5-year all-cause mortality in HD patients.

## Supplementary Material

Supplemental Material

Supplemental Material

Supplemental Material

Supplemental Material
