# Supplementary material for: Iron, coronary artery calcification, and mortality in patients undergoing hemodialysis
Source: Ren Fail. 2021 Feb 18;43(1):371–80. doi: 10.1080/0886022X.2021.1880937 (PMC7894440; doi:10.1080/0886022X.2021.1880937)
Supplement: Supplemental Material [file IRNF_A_1880937_SM7537.pdf]

Supplementary Table: Predictors of 5-year all-cause mortality in hemodialysis patients, according to Cox proportional hazard analyses, and using the dose of all phosphate binders as a variable

Variables	Multivariate analyses								
	Model 1			Model 2			Model 3		
	HR	95% CI	*P*	HR	95% CI	*P*	HR	95% CI	*P*
Age (years)	1.06	1.03–1.10	<0.01	1.06	1.02–1.10	<0.001	1.06	1.02–1.10	<0.001
Male sex	1.02	0.53–1.93	0.94	1.03	0.55–1.98	0.93	1.10	0.58–2.13	0.77
Smoking	1.20	0.61–2.37	0.59	1.34	0.68–2.67	0.40	1.36	0.69–2.70	0.38
Presence of diabetes mellitus	1.30	0.66–2.53	0.45	1.29	0.66–2.50	0.46	1.11	0.56–2.16	0.76
Duration of dialysis (months)	1.00	0.99–1.00	0.32	1.00	0.99–1.00	0.24	1.00	0.99–1.00	0.38
CACS	1.01	1.00–1.01	<0.0001	1.01	1.00–1.01	<0.0001	1.01	1.00–1.01	<0.0001
Serum albumin (g/dL)	0.63	0.29–1.37	0.24	0.78	0.35–1.74	0.53	0.58	0.26–1.28	0.17
Serum CRP (g/dL)	1.67	1.22–2.29	<0.01	1.62	1.15–2.19	<0.01	1.68	1.19–2.26	<0.01
Log serum intact FGF23 (pg/mL)	0.95	0.64–1.42	0.82	0.90	0.60–1.34	0.60	0.90	0.62–1.33	0.60
Erythropoietin responsiveness index (unit/kg/g/dL)	1.03	1.01–1.06	<0.01	1.03	1.01–1.05	<0.05	1.05	1.02–1.08	<0.01
Dose of all phosphate binders (g/day)*	0.51	0.28–0.93	<0.05	0.53	0.28–0.94	<0.05	0.53	0.27–0.94	<0.05
TSAT ≥17%	0.55	0.32–0.97	<0.05						
Fe ≥63 μg/dL				0.54	0.30–0.97	<0.05			
Ferritin ≥200 ng/mL							0.27	0.06–0.87	<0.05

CACS: coronary artery calcification score, CRP: C-reactive protein, FGF23: fibroblast growth factor 23, TSAT: transferrin saturation, Fe: serum iron, Ferritin: serum ferritin, Erythropoietin responsiveness index was defined as the average weekly erythropoiesis stimulating agent (ESA) dose divided by dry weight and average blood hemoglobin. *The prescribed doses of phosphate-binding agents were converted to daily defined doses (DDDs), using the conversion factors provided by the World Health Organization Drug Classification (http://www.whocc.no/atcddd/), and they are expressed as prescribed weekly dose/7 DDD value. The phosphate binders used were calcium carbonate (DDD: 3 g), sevelamer (DDD: 6.4 g), and lanthanum carbonate (DDD: 2.25 g), and other phosphate binders were not prescribed at our institution in 2012.

HR: hazard ratio, CI: confidence interval, For continuous variables, the HRs were computed per unit increase.
