# Supplementary figures and images for: Iron, coronary artery calcification, and mortality in patients undergoing hemodialysis
Source: Ren Fail. 2021 Feb 18;43(1):371–80. doi: 10.1080/0886022X.2021.1880937 (PMC7894440; doi:10.1080/0886022X.2021.1880937)

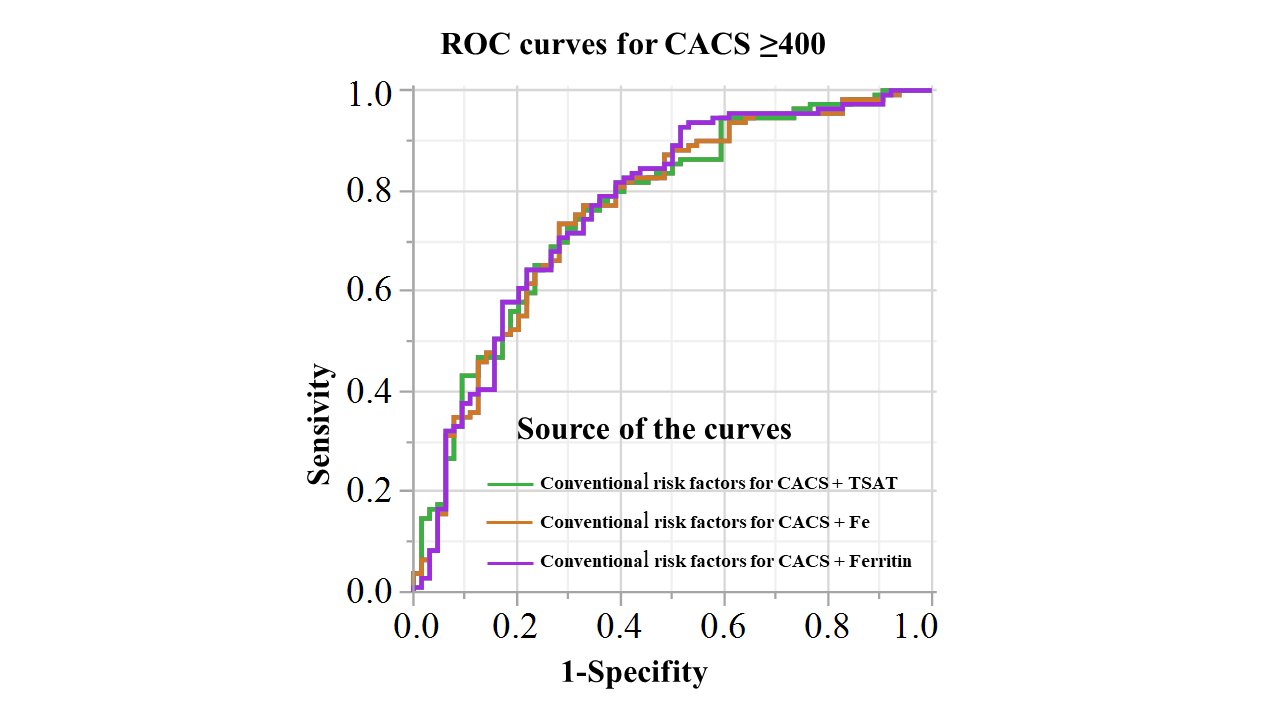

Supplement: Supplemental Material [file IRNF_A_1880937_SM7531.tif]

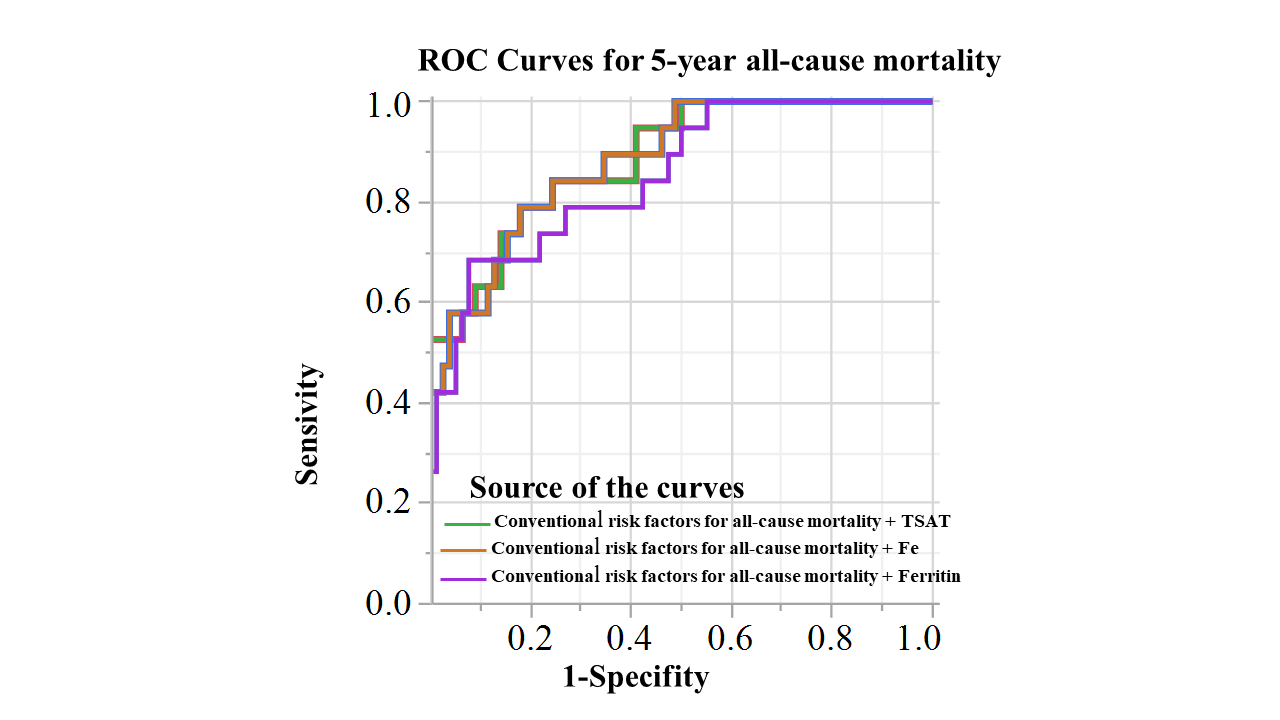

Supplement: Supplemental Material [file IRNF_A_1880937_SM7468.tif]

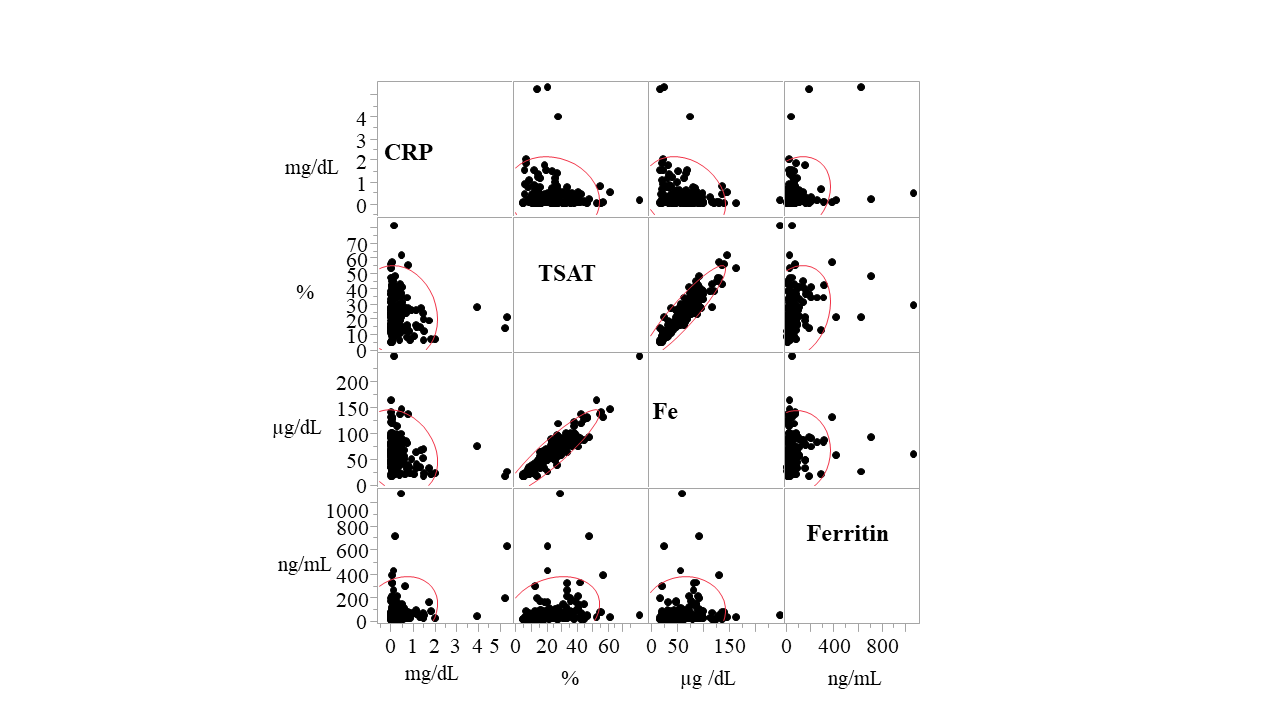

Supplement: Supplemental Material [file IRNF_A_1880937_SM7405.tif]
